# Abstracts and Gleanings

**Published:** 1881-09-20

**Authors:** 


### ABSTRACTS AND GLEANINGS.

**Case of Croup Treated by Passing Catheters into the Trachea by the Mouth.**—In the British Medical Journal for July 24 and 31, 1880, are two papers by Dr. Macewen, on the value of Tracheal Tubes introduced by the Mouth in Edema Glottidis, etc. The cases he records are all in adults. I am not aware that this treatment has been used in children, but its simplicity and advantages are so great that a few notes of a case of croup in which catheters were used may be interesting.

H. J., aged three years and ten months, had measles, the rash appearing on February 15, 1881. On the disappearance of the rash a hard cough supervened, which gradually increased in severity until March 1st. On that date I found him, at 1.30 a.m., suffering from intense dyspnœa, quite unable to speak, and his lips of a dark livid color. His cough was constant, brassy, and without expectoration. The respirations were 35 per minnte, the cartilages of the ribs and sternum being drawn in at every effort to breathe, and crepitation existing over both lungs. The fauces were healthy. The pulse was 144, very weak. Having a No. 11 prostatic catheter with me, I determined to pass it into the trachea instead of performing tracheotomy. Watching an opportunity, while the tongue was depressed with a spoon, the catheter, curved a little more than usual, was passed into the trachea during an attempted inspiration and without the slightest difficulty. A severe struggle followed, lasting perhaps a minute or two, the face becoming purple and the eyes staring with fully dilated pupils. The paroxysmal effort to expel the tube being unsuccessful, a pretty full inspiration partly through the tube and partly through the larynx, followed; about two ounces of frothy, bloody, and purulent mucus were ejected by the tube and the mouth; the livid color disappeared, and he lay down breathing easily through the tube. The presence of the tube did not prevent his swallowing milk, though sometimes a little of this was ejected from it during a cough. The tube was retained in situ by a strip of plaster, and the teeth were prevented from closing on it by means of a pear-shaped piece of hard wood.

Six hours afterward he was much easier, and could say "Yes" and "No" distinctly. The cough continued at intervals of ten minutes, and did not seem altered in character by the presence of the tube. Crepitation still existed over both lungs, an abundant muco-purulent secretion passing both by the tube and the mouth. Hitherto he had been kept in a warm room, but now a bronchitis-kettle maintained a moist temperature of 70° F. The tube was removed without any inconvenience after it had been in the trachea for eleven hours, as he had bitten it, and no air was passing through it. Shortly after its removal symptoms of obstruction gradually reappeared. During the same evening another ordinary gum-elastic catheter No. 12 was introduced, a slight momentary struggle and cough supervening. The presence of the tube led again to a very free expectoration of mucus. In the course of a few hours the respirations and pulse became lower, and crepitation and dyspnœa ceased. When the tube had been in for forty-eight hours and a half it was removed and not again introduced. On March 8th the voice and chest sounds were normal, and he was not seen after the 10th.

This case was a severe one and would have soon ended fatally, had no operation been performed. Tracheotomy seemed inadmissible, neither the case nor the surroundings being favorable for it. *Prima facie*, it would be expected that the introduction of a tube into the trachea of a child against its will would not be so easy as in a consenting adult. That may be so; but it is certain that the operation is extremely easy and simple, and does not take more than two or three seconds from touching the tongue with the spoon till the tube is in the trachea. Had tracheotomy been performed successfully, when would the child have been out of danger? Certainly not so soon as here recorded; for at the end of the third day the child was so well as to be able to breathe freely without the tube, and was quite well before the tenth day after the operation.—*Louisville Med. News.*

**What is Pyæmia?**—In view of the mooted question in the President's case, that is, whether he has been suffering from septicæmia or pyæmia, the following points in the differential diagnosis of these conditions, collected by the Medical Record, will be read with interest:

According to Delfield, under the name of pyæmia are commonly understood several different conditions, giving rise to different lesions.

1. Septicæmia—In this some portion of the body is in a condition of gangrene; that is, the tissues are not only dead, but decomposing, with the evolution of gases, the softening and liquification of the solid parts, and the development of minute organisms, either animal or vegetable. The gangrenous fluids thus produced are apparently absorbed by the lymphatics and blood-vessels, and are thus able to produce marked symptoms during life, and to produce death.

2. Simple Pyæmia.—Persons who have suppurating wounds or abscesses may, without much change in the wound or abscess, be seized with rigors followed by fever, become jaundiced, and die.

3. Metastatic Pyæmia.—This is a very different condition from the other two and may be accompanied with marked lesions. By the agency of various changes about the wound, substances are absorbed which produce in various parts of the system multiple abscesses and hemorrhagic infarctions.

*Agnew.*—The fevers that follow wounds are divided into three kinds:

1. Simple traumatic infective fever.
2. Secondary traumatic fever.
3. Complicated traumatic infective fever.

The secondary traumatic fever may imitate a hectic fever.

The complicated traumatic infective fever includes what are generally known as septicæmia and pyæmia. But Agnew discards the term pyæmia.

*Billroth* classifies the fevers following wounds into—

1. Primary traumatic fever.
2. Secondary traumatic fever.
3. Septicæmia.
4. Pyæmia.

By septicæmia he understands a constitutional acute disease which is due to the absorption of various putrid substances into the blood, which is thereby spoiled so that it cannot fulfill its physiological function.

Pyæmia is a disease which we suppose to be due to the absorption of pus or its constituents into the blood. It is symptomologically characterized by intermittent attacks of fever, etc., and in its pathological anatomy by abscesses and diffuse inflammations. It is clinically distinct from the other varieties.

*Fordyce Baker* believes septicæmia and pyæmia to be distinct clinically, and probably, distinct pathologically. There are more evidences of cerebral disturbance, more diarrhœa, fever, chills, and the symptoms come on earlier in septicæmia than in pyæmia.

*Greenfield* considers pyæmia and septicæmia to be distinct, but attributes both to an invasion of the system by microscopic organisms.

*Erichsen.*—Pyæmia is a name applied to a group of pathological conditions. These include (Virchow) (1) leucocytosis, (2) the formation of thrombi with resulting emboli and abscesses, and (3) an absorption of ichorous matter producing the condition known as septicæmia.

*Bryant.*—According to this author, traumatic fever, septicæmia and pyæmia are all names for one condition, viz., blood-poisoning. These different forms of blood-poisoning differ in degree, not in kind. Traumatic fever may pass into septicæmia, and septicæmia into pyæmia.

The variety of statement and of definition in the above, indicates less real discrepancy than would appear. Some of the authorities quoted are antiquated, while the more modern ones are substantially agreed in fact, if not in letter. The view that what used to be known, and still is recognized by some as pyæmia, is only a form of septicæmia, is the one generally held by modern pathologists. Pyæmia is an intenser form of septicæmia, having clinical and morphological characters of its own. The word pyæmia, however, ought to be abandoned, for its etymology conveys a wrong idea of the real pathology of the disease.

Most surgical writers have little claim to speak authoritatively on pathological points. It is note worthy that Hamilton and Agnew, in their "Surgeries," come nearer to accuracy than the others that we have quoted.

**Can we Make a Positive Diagnosis of Pregnancy Previous to the Occurrence of the Audible Sounds of the Fœtal Heart and the Detection of the Fœtal Movements?**—The subject of an article by Dr. Joseph Tabor Johnson, of Washington, D.C.; read before a Section of American Medical Association, May 4, 1881. It is claimed that in the softened condition of the cervix uteri and the pinkish color and increased temperature of the vagina, we have quite positive diagnostic evidence of pregnancy. It is admitted that the only positive and indisputable signs are determined by auscultation, ballottement and fœtal movements; but these signs are not usually present in the first half of pregnancy. The presence of kiestine in the urine, milk in the breast, the odor of vernix caseosa upon the finger as it is withdrawn from the vagina after a digital examination, the smooth condition of the interior wall of the vagina and anterior cul de sac, associated with a pinkish purple color of the vaginal mucous membrane, the placental souffle, the existence of *gravidence* in the urine, the presence of certain caseous elements resembling milk in the urine, were all passed in review as diagnostic signs of pregnancy; but no definitely stated conclusion was arrived at.

Dr. R. Beverly Cole, of San Francisco, California, did not hear the whole of Dr. Johnson's paper, but thought from what he did hear, it was rather an interrogation than a treatise. He then stated that there were three physical signs of pregnancy which he relied chiefly upon, viz.:

1. Placental souffle, 2d. pulsation of the cord, and 3d. the sounds of the fœtal heart. He regarded the last as the best and most reliable of them all. The pulsations vary from 110 to 140—double of that of the mother. He described the sound as resembling the ticking of a watch under a pillow. Dr. Cole thought no signs positive enough (generally) to justify one in giving decided opinions when consulted on this point. He then cited a case, that of a girl who came to him and wished to ascertain whether or not she was pregnant. She appeared to be about six months advanced. Placental souffle was present, but no pulsation. The navel was protruding—admitted that she had intercourse with a man six months before, and had not menstruated since. The doctor then made an appointment for another examination, but she went to another physician, who examined her six times, called in some three or four others in consultation, who advised the introduction of a sound. This was done, and when it was withdrawn the discharge proved that it had penetrated into an abscess, which, Dr. Cole thought, was undoubtedly due to pelvic cellulitis.

Dr. Albert H. Smith, of Philadelphia, thought placental souffle the most unreliable of signs mentioned by Dr. Cole. He was anxious, he said, to hear Dr. Jos. Tabor Johnson's results from the thermometric observations on the cervix uteri. He thought Dr. Johnson's suggestions in regard to the application of the telephonic principle to the uterine sound, and the use of the electric light for illuminating the vagina, very good—quite brilliant, he would say. Liked the bi-manual method best of all.

Dr. Paul F. Munde, of New York city, agreed with Dr. Smith. His favorite method was the bi-manual. He thought Dr. Smith had touched the key-note in making this statement. He thought that this method, taken with the other signs usually associated, would enable one to make out a case better than by any other methods he knew of. He cited a case in support of his position.

Dr. Alex. Dunlap, of Ohio, agreed with Drs. A. H. Smith and Joseph Tabor Johnson. His method was the same, the bi-manual. The presence of fibroids may sometimes mislead as they enlarge the womb, but they are generally hard when small. Sometimes soft and dropsical when large, and rarely symmetrical. These points he thought it well to notice. Sanious discharge from the os is strong evidence of intra-uterine fibroid.—*Medical Bi-Weekly.*

**Hypodermic Injection of Quinia.**—In the pernicious or congestive forms of malarial fever which Southern practitioners so often see on the water courses, the matter of quick recognition is hardly more important than the method of administering the needed medicine.

Take a typical case of this fever in the comatose stage. You will find pulmonary congestion, with accumulation of bronchial mucus. The pulse is small and rapid; the surface bathed with cold sweat; breathing thirty to forty in the minute with deep sighing at intervals; evacuations of the bladder and bowels are unconsciously performed; the patient is restless; the jaws are generally firmly closed; the mouth filled with viscid mucus; the tongue pale and coated with thick white fur; the pupils permanent; the eyes partially closed. To complete the dangerous aspect of the patient the temperature reveals 103° to 106° Fahrenheit.

In such a case as this, every minute is of importance. There is no doubt as to what the patient ought to have. It is too well recognized that opium, alcohol and quinia, one or all, are imperatively demanded, especially quinia. But what is the use in putting quinine into the mouth of a patient whose power of absorption is as feeble as in this case? You want cinchonism at the earliest hour possible, and your quinia may remain inactive in the stomach, and finally be rejected.

The old plan of denuding the abdomen by means of Granville's stronger lotion, and applying quinine in solution over the surface saved many lives. But the objections to this process was that it left the patient with a large and sometimes indolent sore.

When the attention of the profession was called to the hypodermic use of quinine, the idea was seized upon as exactly the thing for these rapidly fatal cases of pernicious malarial fever. It was soon discovered that undissolved quinia flowed badly through the hypodermic needle, and to dissolve it in enough acid to make a clear solution, the result was almost sure to be an abscess. We remember several cases in which deep and ugly ulcers were caused by the solution of quinia in sulphuric acid.

The writer saw recently a case of pernicious malarial fever contracted in the river swamps. The patient was brought to the city from a long distance across the ferries in the hottest part of a July day. His condition was about as we have already enumerated the symptoms.

There was no hesitation as to what to give the patient, if indeed anything in his desperate condition could avail him. It was agreed to give quinine in solution with hydrobromic acid—40 grains quinine to a dilute solution of equal parts of hydrobromic acid and water ʒij each—hypodermically. Four separate insertions were made, over the abdomen, the thighs and buttocks.

Most profound cinchonism ensued in a short time, and the patient was well enough the next morning to be sent to the hospital. His recovery was rapid, and there was not the slightest indication of inflammation about any of the punctures. Successful hypodermic injections of quinia are not so rare as to attract our attention as novelties, but as rational a means as the administration of quinia hypodermically is, it is being abandoned, because of the bad ulcers resulting from it, and it needs the records of successful cases to reassure the profession as to its propriety. It is beyond question that nothing at our command can be relied upon as confidently as the hypodermic use of quinia in pernicious malarial fever.—*N. C. Med. Journal.*

**The Therapeutical Indications of Bromide of Ammonium.**—Dr. E. Halsey Wood, of Hersey, Michigan, in a communication to the Michigan Medical News, April 25th, 1881, first draws attention to the fact that most authorities, when referring to the bromides, are thinking only of the potassium salt, and regard the effect of the different bromides as analogous. After showing this to be an error, he says:

The following are the symptoms which I have found to indicate the use of the bromide of ammonium: Frontal headache, suffusion and blurring of the eyes (asthenopia), soreness of the upper lids, ptosis or convulsive closure of the eyes due to paresis of levatores palpebrarum, a sense of swollenness or bulging in the eyes (exophthalmos), a wavering dilatation of the pupil, injected and icteroid conjunctivæ, excessive sweating, sleeplessness, disagreeable dreams (phantasmagoria), anorexia, nausea, vomiting and retching, stiffness of the fingers, numbness, swollenness, and sweating of hands, blue or other abnormal color under the nails, especially congestion of hands, pain in nape of the neck running up into the occiput, pain in shoulders, elbows, and knees, pain at sacro-spinal junction (lame back), a sense of weariness (muscular debility) or pain, or pain with spasm (cramps) in the calves of the legs, pain between the shoulders and under scapulæ, any degree of coolness of hands or feet appreciable by the normal sense or caloric, œdema of feet and ankles, congestive, intestinal, uterine, renal, gastric and pulmonic hemorrhagos, despondency and irascibility, lassitude, languor, listlessness or weariness, hyperthermy and hypothermy, hot flashes and cold chills, hopelessness (acute formapathy), drowsiness in the day-time, atonic voice, tinnitus aurium due to congestion of the labyrinth, pulse slow or thready, a sense of chilliness, and a sense of constriction around the chest.

In the same journal, May 10th, he relates several cases illustrative of its value in the treatment of cholera. He employs it in full doses frequently repeated (adults gr. xx, every five minutes), according to the urgency of the case. As to the *modus operandi*, he says:

The remedy acts as a sthenic; it energizes the ganglia and restores innervation, and all the evidences of deranged function disappear under its influence. It is as specific in the mild as it is in the severe degree of gangliasthenia, and thus not only exhibits its potency but proves that the shape of disease assumed is due to different degrees of the same condition.—*Med. and Surg. Rep.*

**Eucalyptus.**—Eucalyptus is recommended in all catarrhal affections of the air passages, from the common coryza to chronic bronchitis, and of the alimentary canal, ulcers in the stomach, chronic diarrhœa, and in the diseases of the urinary tract, such as inflammation and irritation of the bladder, gonorrhœa and gleet. I have found it of special benefit in irritable bladder. One case that lately came to me from Fountain county, Indiana, in which belladonna and camphor relieved her perfectly, while in this city, but on her return home the irritability came on with double force, eucalyptus gave immediate relief. She being a very intelligent lady, I will copy a portion of her letter, dated Dec. 4th, 1880:

"I am quite free from distress and able to work the early part of the day, but about three in the afternoon the "ache" begins; there is a constant desire to pass water, and a strained, protruding feeling at the neck of the bladder; then follows a fearful itching, scalding, aching sensation, which well nigh drives me frantic.

"After a time of suffering, which is exhausting, the distress subsides. I eat heartily, but so bruised and beaten do I feel that I can only take the edge of a chair for rest. I have such a time, also, every night, being obliged to get up every few minutes to pass a few drops of water, and the passage is often agonizing. There seems to be about the same quantity, though at times it is strong and high-colored."

We have here pure irritability, and half-drachm doses of fluid extract of eucalyptus quickly controlled the trouble, as she states in a letter dated December 22:

"I have found out the benefits of eucalyptus in my case. Its relief came most opportunely, for I don't know how I could much longer have endured the terrible strain of the bladder difficulty, and I am so happy to find a balm for that," etc., etc.

I have obtained good results, also, in several cases of incontinence of urine, and in some cases of gonorrhœa I consider it superior to copabia and the oil of sandal wood. It seems to relieve the pain and scalding more promptly, and reduce the discharge sooner than they.

I have seen good results in chronic bronchitis from its use. I usually use the fluid extract for urinary troubles, and the tincture for malarial fevers and bronchitis. Dose of each, about half a drachm.—*Dr. H. A. Foster, in Phys. aud Surg. Inv.*

**Hepatomy for Hydatids.**—Lawson Tait, F. R. C. S., in British Medical Journal, writes: The sixth case of this operation, which I have performed, like the others, has been remarkable for the speedy and complete recovery of the patient.

A. M. S., æt. 7, early in May last, suffering from severe symptoms due to a tumor on the right side, and above the level of the umbilicus, which was clearly cystic, and, in all probability, connected with the liver. It gave great pain, and I diagnosed it to be a hydatid tumor of the liver. The child had always been regarded as delicate. A year ago, her mother noticed that her motions were rather white-colored. Swelling was noticed in abdomen about November last, and she complained of pain across the back and shoulders. December, 1880, there was a firm tumor just below the ensiform cartilage, the dullness extending round the side. In February, there were some nodules on the surface of the liver; also tumor was more movable.

When admitted, had a tumor about the size of a fœtal head, which was extremely tender to the touch. The child was very sick, and appearance warranted interferance. Opened the abdomen May 20, making an incision about three inches long, one and a half inch to the left of the umbilicus, the lower end corresponding to the umbilical level. When the cavity was opened, it was perfectly clear that the tumor was situated in the liver, and was a hydatid cyst. Removed from it, by means of an aspirator, about twenty-six ounces of clear fluid, containing a large number of scolices. Then enlarged the aperture in the liver to about one and a half inch, and secured its edges to the edges of the parietal wound by means of a continuous suture, and fastened in a wide, soft, India-rubber drainage tube about six inches long. She went on perfectly well; severe symptoms immediately relieved, and May 26 the mother-cyst came away entire. Drainage-tube removed May 30; and June 2 she left with the wound quite healed, having gained greatly in weight, and having acquired a perfectly healthy appearance. No attempt was made to conduct the case upon Listerian principles, the only dressings used to the wound being red lotion and absorbent wool.

**On the Treatment of Gonorrhœa.**—Dr. A. W. Morris, of Kentucky, in Medical Herald, says: Early after I graduated, I was disappointed in the treatment of this disease, both by astringent injections and the internal administration of remedies; and, as I had a large number of cases coming to me, I made an effort to secure a treatment giving more satisfactory results. In my series of experiments, I purchased a "Bartholow's Catheter," an instrument with an olive bulb on on the point, and holes in the shoulder of the bulb—the point not being pierced. The tube being the size of a No. 6 catheter, the bulb being much larger, presenting an onward flow of the injected fluid, and causing it to flow backward and outward. I attached this to a good "pump syringe," by rubber tubing, and the next case I treated gratuitously, for the privilege of using my new machine. After throwing in about a gallon of cold water, I took a small penis syringe, and gave an injection of sulphate of zinc, as thorough as possible, and told my patient to call again next day. I saw no more of him for a month, and then "blew him up" for not coming around and taking more of the treatment. He replied, that as it cured him, he thought there was no use, and never thought any more about it. Holding the theory of the limitation of the disease, as given by Bumstead, and other authorities, I had no faith in the result of this case, and determined to give it a further test. I reached the following result, astonishing as it may appear, nevertheless it is true: Out of 25 cases, 22 were entirely well 24 hours after the treatment. No discharge, and no treatment of any kind was given, other than washing out the urethra, and the sulphate of zinc injection first and once only given. One was well in three days, one in seven days, and the other, a drinking man, who kept up his whoring all the time, was cured in two weeks. After this, in fifteen cases, the result was not so satisfactory, but much more than the old treatment.

**Euonymus Atropurpureus** (**Wahoo**).—I notice in the January Gazette an article on "Euonymus At.," from Prof. Henning, which I endorse. Its effects on the liver are as certain as those of "irisin," and more persistent, acting as a sure deobstruent.

I have used the remedy, and have rarely been without it since 1851. It is one of the best remedies in the materia medica, when used in small doses, for certain indications, which are torpidity of the mucous membrane and liver, for hemorrhoids with torpidity of the peristaltic action of bowels, and in the erysipelatous diathesis. Consequently it gives tone to the stomach and digestive functions. It stimulates secretion from liver and blood into the bowels, and perhaps cures hemorrhoids through such action, and in this action proves one of our best alteratives. It is as sure a remedy for hemorrhoids as any I ever used internally, leaving the bowels in a soluble condition after discontinuing its use. I think it superior to cascara sagrada for overcoming habitual constipation, for this reason: When the action (normal) becomes established, its use can be dispensed with, the bowels continuing to move regularly for a long time after, provided you obtain its full effect upon the liver and bowels, and their secretions fully established. I have cured some of the worst cases of hemorrhoids with this single remedy, and but few doses, they being small and often repeated. I find the euonymus Amer. to be equal in its effects to the euonymus atropurpureus, and either a special tool for the vital force to use in the certain conditions named.

Suffice it to say, it is a positive remedy, and should never be left out of our direct remedies, or its direct action forgotten by reliable therapeutists who study the action of remedies in the system, and the conditions requiring them.—Dr. Woodward in *Ther. Gazette.*

**Sanguis Bovinus Exsiccatus.**—Dr. O. J. Wesley, of Bainbridge, N. Y., in the Gazette, says: Below I beg to give particulars of a case in which I used the new preparation, "desiccated blood,' with good results.

Mrs. A. B. aged 45, American, mother of two children, suffering from infiltrating cancer of some six months' duration, which was removed December 9th, 1880, under the influence of chloroform. After operation, hypodermic injection of one-fifth grain morphia was used, which produced extreme nausea, lasting between thirty-six to forty-eight hours, causing extreme straining and re-opening of wound, resulting in great loss of blood before assistance arrived. Upon my arrival I found the patient in an almost bloodless condition, and unable to retain anything upon her stomach with the exception of minute particles of ice. I administered beef tea, prepared from fresh beef, per rectum, with only partial benefit, and having had a sample of desiccated blood left me, prepared it as directed and administered two ounces at once, giving directions for further injection of the same quantity every two hours. Upon my calling next day I was more than pleased with the change apparent. The patient's countenance had altered trom a dull leaden hue to one nearly approaching that of health, and she reported herself as feeling stronger and better in every way. She continued the use of the blood until the stomach was able to assimilate food, and is now able to move around as before operation.

**Trichlorophenol, a New Antiferment.**—This compound is described as a more powerful antiferment than carbolic acid. We learn from Prof. Stadler's Chemical Notes in the American Journal of Pharmacy, that Mr. Dianin, as reported at the annual meeting of the Russian naturalists, held at St. Petersburg, Jan. 1880, found that on mixing solutions of phenol (carbolic acid) and "chloride of lime," a reaction at once sets in and the chief product was trichlorophenol. This compound was also found to arrest fermentation in a much greater degree than phenol itself, and the mixture above mentioned was therefore recommended by him as much better suited for application to sloughing wounds than phenol itself.

The matter has since been thoroughly studied by C. O. Cech. He prepared the chlorophenols by the direct action of chlorine upon phenol The crude product was a blood-red crystaline mass of strong odor and burning taste, easily soluble in ether, and is much less caustic even in this condition than carbolic acid. By repeated pressings of the crystaline mass between filtered paper it is obtained in lustrous white crystals, soluble in ether and precipitable from alcoholic solution by the addition of water in the form of white flakes. The alcoholic solution can be used conveniently for saturating bandages for direct application to wounds.—*The Druggist.*

**Noma, or Gangrene of the Mouth.**—Noma generally begins by the appearance of a patch of induration situated on the mucous surface of the cheek near the labial commissure, and which is quickly surrounded by minute phlyctenulæ. The neighboring parts swell, the patch becomes black, it spreads on the surface and deeply, the soft tissues become involved, and even the bone is affected. After the removal of the sphacelated portions a hideous hole remains in the side of the cheek. Death occurs in seventy cases out of one hundred. In case of cure, extreme disfigurement, with adherent cicatrices, is apt to ensue.

This disease has sometimes been considered to originate in some disorder of the nervous system, particularly the vaso-motors of the face. Krasine, however, is inclined to think that it is due to a cutting off of the blood-supply in an anæmic and broken-down person by the exercise of pressure. This pressure may, in some cases, be the result of lying on one side or the other during a prolonged illness, and is thus nothing more than a gangrene from decubitus.

Noma is generally limited to one side of the face; it rarely attacks the other side. Above, it may reach to the free border of the under eyelid and to the ear. It rarely passes beyond the border of the lower jaw. The tongue and the eye of the affected side remain untouched.

Noma attacks children because, in Krasine's opinion, the amount of blood in the body is relatively smaller than in adults, nutrition changes are active, and anaemia is quickly produced and has grave consequences. Why the disease should attack little girls by preference is as yet inexplicable.

The treatment of noma has hitherto been by means of local remedies, caustics, the cautery, etc. Krasine, however, speaking from his point of view of the origin of the disease, urges improved nutrition, tonics, stimulants, etc., with simple antiseptic dressing.—*Medical Times.*

**Viburnum Prunifolium in Threatened Abortion.**—J. K. Milbourne, M. D., in Therapeutic Gazette says: I beg leave to report what I consider a remarkable effect of viburnum prunifolium:

At 11 a. m., April 19th, was called to see Mrs. W. Found her suffering with a severe attack of pneumonia of the left lung.

She was in her seventh month of gestation. Severe lumbar pains. I recognized that the only chance to save my patient was to prevent abortion. Having but little confidence in the old remedies, under such conditions, I determined to try the new. Was compelled to send for it and did not receive it until 8 p. m., when the pains were coming on regularly every five minutes, with considerable "show." I commenced giving teaspoonful doses of the fluid extract viburnum prunifolium every hour. After the third dose all contractions ceased. I then ordered it every three hours for 24 hours. In the meantime, it might be mentioned, I gave for the pneumonia, thirty grain doses of quinia twice a day, which drug is, it is known, claimed by some authors to be an oxytocic. Since the contractions ceased the viburnum has been master of the situation. The patient is now discharged as well.

If this drug proves as useful in all cases as in this, it is invaluable. I can give it the whole credit in the case reported, for I used nothing else for that purpose.

**The Use of the Lancet.**—I have a bit of a confession to make, and also a request. I confess being so much of an old fogy, that for twenty years I had carried the deadly thumb lance in my pocket, and what hurts me most, have not used it as often as I ought. During the four years of my pupilage, I saw my preceptor use it once only. For two years after graduating I did not use it at all. Since then I have used it more and more each year, and can truthfully say, that I have never seen a single bad effect from its use; but have often had to lament in sack-cloth and ashes that the prejudice of patients, or the opposition of consultant, has compelled me to witness many a death which might have been avoided, and many a long convalescence which might have been shortened, by an early and free use of the lancet. My fogyism extends even to the use of calomel in many acute inflammatory diseases. The request mentioned is, that some vigorous writer (or rather thinker) would do for calomel what Dr. Corson is doing for the lancet. It is a belief that fire will never melt out of me, that a proper use of these two agencies would completely cure many men and more women of acute inflammations, who, under the expectant and dish-water treatment, only so far recover as to be thereafter daily reminded that it has left them a legacy of which they cannot dispose. In the Reporter of April 30th, Dr. Stinson asks : "When bleeding is indicated, what practitioner will refuse to resort to the lancet?"

Why, Doctor, that is just the very point. Nineteen out of twenty fail to see the indication; and the chances are vastly in favor of the twentieth one being afraid to act in opposition to the *great minds* of the profession.—F, R. Millard, M.D., in *Med. and Surg. Reporter.*

**An Anæsthetic Car.**—A correspondent writes to an English contemporary:

"When I was in Paris I had an opportunity of seeing Paul Bert's anæsthetic car at work. I was at the St. Louis Hospital one morning; the car was there, and some operations were to be done on it. We—the patient, doctor, and his students—went into the car; the door, air-tight, was closed, and air forced into the car; in a few minutes my ears began to feel strange, and I was told to swallow, yawn and blow my nose, which I did every few minutes, and so made the pressure equal on both sides of the drum of my ears. The patient laid himself down on the operating table, and the anæsthetic agent was given him. He took it very quietly, did not struggle, and was soon insensible. While he was unconscious an epithelioma was removed from his lower lip; after the wound was sewn up, the compressed air was allowed to escape; the patient got up from the table, walked out of the car, and lay down on the grass; he complained of no headache nor nausea, but said he felt just as usual. The car is on wheels, and is carried from hospital to hospital; the hospitals being under government, the car is a public one, and is taken all over Paris.—*Med. and Surg. Rep.*

**Opium Habit.**—Dr. Gray, in Medical Brief, says: In erythroxyion coca we have a remedy which I am positive will cure the great majority of the victims of opium, but the physician must experiment a little in each case so as to arrive at the proper dose needed by his patient. This remedy certainly does so far exalt and support the powers of the whole organization, that the great depression following the gradual or even sudden withdrawal of the accustomed stimulant is not felt, or only to a small extent, by the patient.

In using the coca a reliable article should be obtained. Alkalies (sod. bicarb.) taken shortly after the coca increases its exalting and supporting effect. Attention to excretion is also of great importance. Other additional means may be required, and these are of course indicated by the condition of the patient.

**Phenol.**—This purified form of carbolic acid is generally to be preferred. At a meeting of the St. Louis Medical Society, reported in the St. Louis Medical and Surgical Journal, November, 1880, Dr. Carson presented a specimen of pure *phenol*, which he said they had adopted altogether in cases of antiseptic surgery, at least where Lister's method is practiced. It is recommended by Lister himself. It is less irritating, and on account of the odor, not so disagreeable as our ordinary carbolic acid. It causes a little tingling of the ends of the fingers after they have been in the solution for some time, though not nearly so decidedly marked as ordinary carbolic acid. It is expensive, costing four dollars per pound, and it requires considerable time, four or five months, to procure it from the manufacturers.—*Med. and Surg. Reporter.*

**Duboisia and Atropia.**—1. In solutions no stronger than two grains to the ounce, duboisia sulphate is free from danger.

2. That the two-grain solution of duboisia sulphate more rapidly paralyzes the ciliary muscle than a four-grain solution of atropia sulphate.

3. That the duration of its effects is less than half that of atropia sulphate.

4. That the preparations now in the market are more liable to irritate the conjunctiva than neutral solutions of sulphate of atropia.

5. That in the treatment of inflammations of the eye duboisia is quite as useful as atropia, and may therefore be used as a substitute. —*Therapeutic Gazette.*

**Menthol.**—This crystaline solid, derived from oil of peppermint, was brought forward last year, in Edinburg, as an antiseptic and antineuralgic. At present it is too costly for use for the former purpose.—*Med. and Surg. Rep.*

**Relation of Fœtal to Maternal Circulation.**—To determine the extent of communication between the two circulatory systems, Dr. Maas injected into the veins of pregnant rabbits different colored pigments and septic fluids. If the blood of the fœtus was examined from one-half to one hour after the injection, he could always detect in it the above granules, with bacteria and micrococci, similar to those introduced into the maternal circulation. But if the examination was made at a later period they could no longer be found in the blood, but were present in the liver and other organs. These facts are thought to explain why the statements of various observers are so contradictory, and, in some cases, negative.—*Przeglad Ckarski.*

**Sclerotic Acid.**—The constituents of ergot are many and complex. According to the last authentic analysis, there were found eight distinct principles, besides the many inert approximations that go to form the general structure of the *sclerotium.* For a long time ergotin was supposed to be the active ingredient, but it is now disputed in favor of sclerotic acid, which was found among the eight principles in the last analysis. It seems to be in favor with the profession in the East for hypodermic use, for which purpose it seems well adapted. It is reported that it retains its strength for a longer time than ergotin, and does not cause any injurious inflammation at the seat of puncture. The ordinary hypodermic dose is from $\frac{1}{3}$ to $\frac{1}{2}$ grain three times a day.—*Pacific Medical Journal.*

**Gastric Absorption.**—The question of the manner of absorption by the stomach is an open one, to decide which Dr. Anrep undertook a series of carefully and ingeniously conducted experiments. After having made a gastric fistula in a dog, he allowed it to recover entirely from the results of the operation. Then he introduced into the pyloric opening of the stomach, and down into the duodenum, an apparatus resembling Barnes' dilator, completely shutting off the cavity of the stomach from the rest of the alimentary canal. He then placed in the stomach a determined quantity of different substances, and after a lapse of time he examined it to see how much of them was left. He found that sugar was absorbed more quickly than anything.—*Vratch, No.* 46.

**Successful Transplantation of Human Bone.**—The Glasgow Med. Journal informs us that at the meeting of the Pathological and Clinical Society of that city, April 12th 1881, Dr. William Macewen showed a patient on whom transplantation of human bone had been performed, whereby over two-thirds of the shaft of the right humerus had been restored. The grafts were taken from six wedges of bone removed from limbs of patients affected with antero-tibial curves, and were reduced to very small fragments previouly to insertion. The patient was formerly shown to the Society after the first graft had been completed, when there was a restoration of the upper part of the shaft, to the extent of one inch in length. Now, the shaft was completely restored, and the right humerus only measured one-half inch shorter than the left.—*Med. and Surg. Reporter.*

**Caroba, Iodoform and Boracic Acid in Syphilis.**—Annie C., 24, a servant, contracted syphilis four years ago. Under mercurial treatment she seemed to have eradicated the disease, but eight months ago three large rapidly spreading ulcers made their appearance. So fast did they spread and deepen that inside of ten days one of them was more than four inches in diameter, the others but little smaller. Placed her on carobæ (P. D. & Co.), and sprinkled the ulcers every hour with iodoform and boracic acid, equal parts. From the very first application of the iodoform and acid there was a decided improvement in the appearance of the ulcers. Result, a perfect cure, apparently.—D. F. Powell, M. D., Lanesboro, Minn., in *The Medical Summary*.

**Glycerine in Gastric Flatulence, Acidity and Pyrosis.**—Glycerine does not prevent the digestive action of pepsin and hydrochloric acid; hence, while it prevents the formation of wind and acidity, probably by checking fermentation, it in no way hinders digestion. One or two drachms may be taken either before, with, or immediately after food; in water, coffee, tea, or lemon and soda water. In tea and coffee it may replace sugar, a substance which greatly favors flatulence, as indeed, does tea in many cases. In some instances a cure does not occur till the lapse of ten days or a fortnight.—*Kings Co. Proceedings*.

DR. A. E. BURKHARDT has endeavored to ascertain if vaccination of the mother during pregnancy will protect the child from vaccinia, and eventually against variola. The children of six mothers who were vaccinated or revaccinated after the sixth month, with complete or partial success, were vaccinated without effect. The number of experiments was too small to justify a judgment, especially when it is remembered that very young infants are often insusceptible to vaccinia, although they may become so in a few months.—*Pacific Med. Jour.*

**Tartrate of Morphia.**—This salt of morphia is recommended as a suitable one for hypodermic use; because a solution of greater strength can be obtained than with the acetate or sulphate. It is quickly made by dissolving 150 grains of the alkaloid morphia and 22 grains (or a sufficiency) of tartaric acid in one-half ounce of hot distilled water, and evaporating in a moderately warm place.—*Pacific Med. Journal.*

**Eugenol.**—The antiseptic properties of oil of cloves and oil of peppermint have long been known, since they have often been employed to prevent starch, etc., from becoming mouldy. They have also been employed as remedies for toothache. Eugenol, which is extracted from these oils, and which is also known as eugenic or caryophyllic acid, is found to have similar powerful antiseptic properties. It has a formula, $C_{10}\ H_{12}\ O_2$.—*Med. and Surg. Rep.*

**Thymol.**Thymol-vaseline ointment is made by dissolving twenty grains of thymol in one ounce of vaseline. It is useful in eczema and as a parasiticide. It is said that thymol has the property of immediately removing the smell of tobacco.—*Phil. Med. Times.*

